# Spatiotemporal monitoring of water quality degradation and trophic state in a severely polluted tropical urban lake in Southern India

**DOI:** 10.1007/s11356-026-38316-4

**Published:** 2026-10-23

**Authors:** Satyam Shah

**Affiliations:** School of Geography, Geology and the Environment, University of Leicester, University Road, Leicester, LE1 7RH UK

**Keywords:** Water quality monitoring, Chlorophyll-a, Eutrophication, Urban lake pollution, Bellandur Lake, Remote sensing

## Abstract

Bellandur Lake in Bangalore, India represents one of the most severely polluted urban water bodies in South Asia, necessitating systematic monitoring for environmental management. This study employed Sentinel-2 Multispectral Instrument imagery to assess spatiotemporal variations in water quality parameters from January 2023 to December 2025. Six water quality indicators were derived using established remote sensing algorithms: chlorophyll-a (estimated using the Normalised Difference Chlorophyll Index as the primary method), turbidity, total suspended solids, coloured dissolved organic matter, Secchi depth, and floating algae index. A total of 100 Sentinel-2 images were processed using Google Earth Engine, yielding 93 valid observations across 28 months after excluding monsoon periods affected by cloud cover and confining analysis to complete years 2023–2025. Results indicated severely degraded conditions with mean NDCI-based chlorophyll-a of 71.31 µg/L (median 70.24 µg/L), consistently classifying the lake as hypereutrophic. Mann–Kendall trend analysis revealed statistically significant increasing trends in chlorophyll-a (*τ* = 0.144, *p* = 0.041) and floating algae index (*τ* = 0.419, *p* < 0.001), with concurrent significant decreasing trends in turbidity (*p* < 0.001) and total suspended solids (*p* = 0.001). Pixel-wise FDR-corrected trend analysis identified 33.7% of the lake area exhibiting significant increasing chlorophyll-a trends. The lake consistently exceeded Central Pollution Control Board standards, with 100% of observations surpassing permissible total suspended solids limits and 98.9% of observations classified as hypereutrophic. These findings are interpreted as indicative of relative spatial and temporal patterns given the absence of site-specific in situ calibration; however, they provide valuable baseline information for management interventions and demonstrate the utility of satellite remote sensing for monitoring polluted urban water bodies.

**Supplementary Information:**

The online version contains supplementary material available at https://doi.org/10.1007/s11356-026-38316-4.

## Introduction

Urban lakes provide critical ecosystem services including flood mitigation, groundwater recharge, microclimate regulation, biodiversity habitat, and recreational opportunities (Birch and McCaskie 1999; Xia et al. 2025). However, rapid urbanisation coupled with inadequate wastewater management has resulted in severe degradation of urban water bodies globally, particularly in developing countries experiencing accelerated population growth (Carpenter et al. 1998; Dodds et al. 2009). The deterioration of urban lake water quality poses significant risks to public health, aquatic ecosystems, and surrounding communities, necessitating systematic monitoring approaches to inform remediation strategies and evaluate management effectiveness (Smith and Schindler 2009).

Conventional water quality monitoring relies on in situ sampling and laboratory analysis, which, although accurate, presents limitations in spatial coverage, temporal frequency, and cost-effectiveness (Gholizadeh et al. 2016). Remote sensing technologies have emerged as powerful complementary tools, enabling synoptic observations of water quality parameters across extensive areas with regular revisit frequencies (Palmer et al. 2015; Topp et al. 2020). Satellite-based monitoring facilitates detection of spatiotemporal patterns that would remain undetected through conventional point sampling approaches alone.

The Sentinel-2 mission operated by the European Space Agency under the Copernicus Programme provides multispectral imagery at spatial resolutions of 10 to 60 m with a 5-day revisit time at the equator, making it particularly suitable for monitoring inland water bodies (Drusch et al. 2012). The multispectral instrument includes 13 spectral bands, with red-edge bands specifically sensitive to chlorophyll absorption features relevant for aquatic applications (Toming et al. 2016). A rapidly expanding body of research has demonstrated the utility of Sentinel-2 for retrieving water quality parameters in diverse inland and coastal environments (Pahlevan et al. 2020; Bernardo et al. 2023; Wang et al. 2024; Okomo et al. 2025). Recent studies have applied Sentinel-2 for large-scale chlorophyll-a monitoring across hundreds of lakes using standardised NDCI and MCI algorithms (Salls et al. 2024), and regional frameworks combining Sentinel-2 with optical water type classification have been developed to accommodate the diversity of optical conditions across mixed lake systems (Joffre et al. 2026). In Indian water bodies specifically, Google Earth Engine-based Sentinel-2 workflows have been applied to monitor water quality dynamics in lakes and coastal lagoons including Chilika Lake, demonstrating the feasibility of this approach for tropical South Asian systems (Das et al. 2024; Kolluru et al. 2023). Despite these advances, empirical algorithm performance in extreme hypereutrophic conditions such as those found in heavily sewage-impacted urban lakes remains poorly constrained, with documented challenges in multi-constituent optical environments (Adjovu et al. 2025; Jaywant and Arif 2024).

Bellandur Lake, situated in the southeastern region of Bangalore, Karnataka, India, represents one of the largest and most severely polluted urban lakes in South Asia. The lake receives substantial volumes of untreated and partially treated sewage, industrial effluents, and stormwater runoff from the rapidly expanding Bangalore metropolitan area (Ramachandra and Kumar 2008; Mampilamthoda and Chinnasamy 2025). The lake has gained international attention due to recurring incidents of toxic foam formation and occasional surface fires resulting from methane emissions, highlighting extreme eutrophication and organic pollution levels (Das et al. 2023). Despite its ecological significance, systematic long-term multi-parameter monitoring using satellite remote sensing remains limited for this water body.

Previous remote sensing studies of Indian urban lakes have focused primarily on seasonal assessments or single-parameter analyses (Bareuther et al. 2020; Bhateria and Jain 2016). Recent work has begun to leverage Sentinel-2 and Google Earth Engine for multi-parameter water quality assessments in Indian lacustrine and coastal systems (Das et al. 2024; Anand et al. 2024), and machine learning-based retrieval frameworks calibrated against in situ measurements have been applied to Indian inland water bodies including Chilika Lagoon (Kolluru et al. 2023; Maniyar et al. 2023). However, multi-year satellite monitoring of hypereutrophic urban lakes specifically impacted by sewage and industrial effluents remains limited in the Indian context. The relationship between pre-monsoon and post-monsoon water quality conditions, particularly relevant given pronounced monsoon seasonality in peninsular India, has received insufficient attention in remote sensing-based assessments. Understanding these seasonal dynamics is essential for comprehending pollution loading patterns and evaluating the effectiveness of natural flushing processes.

The present study addresses these knowledge gaps by conducting a comprehensive 3-year assessment of water quality dynamics in Bellandur Lake using Sentinel-2 imagery spanning January 2023 to December 2025. The specific objectives are as follows: (1) to characterise spatiotemporal variations in multiple water quality parameters; (2) to identify statistically significant temporal trends at both lake-averaged and pixel-wise spatial scales using FDR-corrected multiple testing procedures; (3) to compare pre-monsoon and post-monsoon water quality conditions; (4) to assess compliance with national water quality standards; and (5) to evaluate the trophic status using the Carlson Trophic State Index based on NDCI-derived chlorophyll-a as the primary indicator. All satellite-derived values are interpreted as indicative of relative spatial and temporal patterns, given the absence of site-specific in situ calibration data.

## Materials and methods

### Study area

**Fig. 1:**
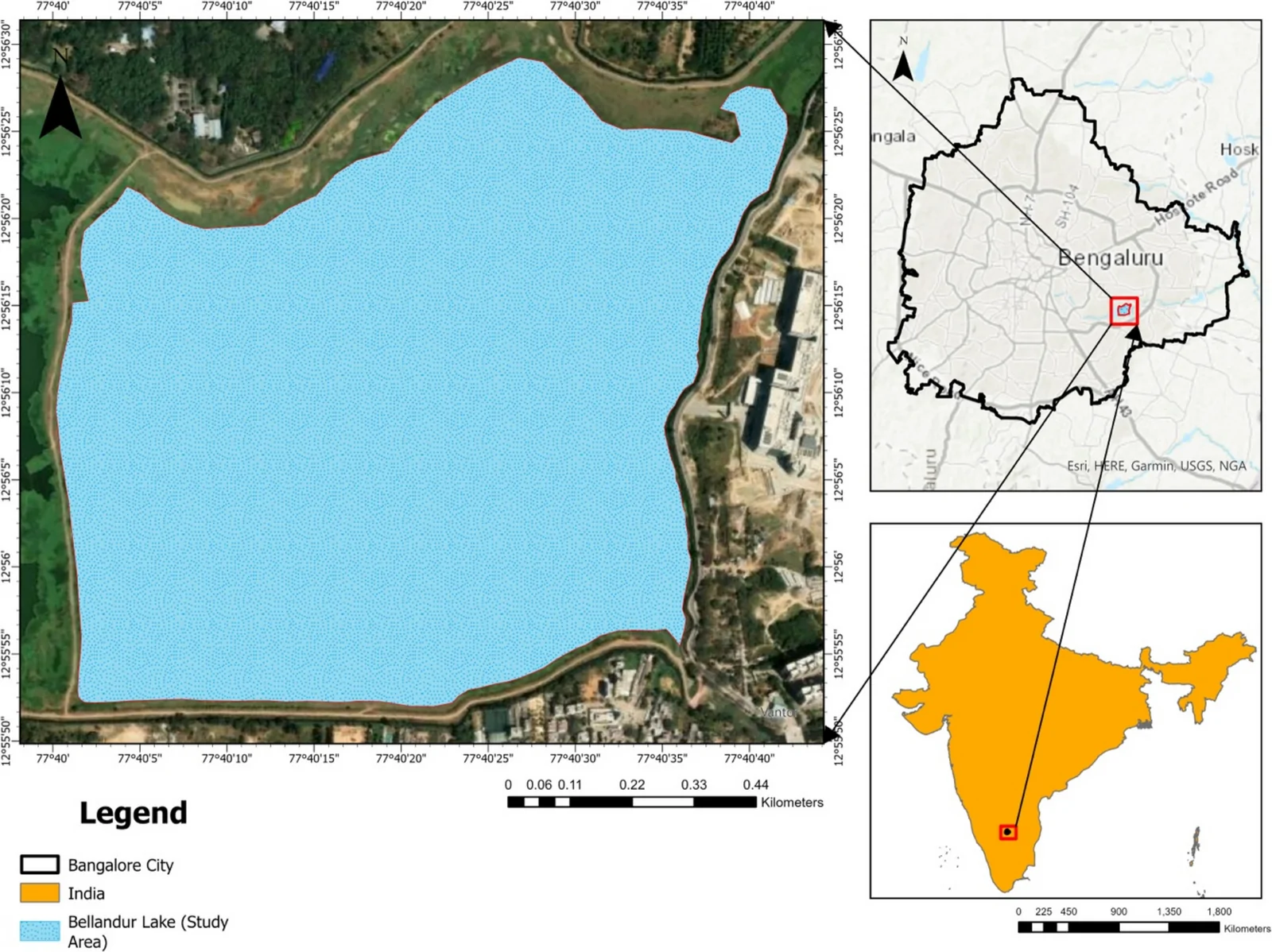
Study area map of Bellandur Lake showing the delineated lake extent and surrounding urban context. Inset maps provide regional and national location within Bengaluru and India

Bellandur Lake is located in the southeastern part of Bangalore, Karnataka, India, with a centroid at latitude 12.936° N and longitude 77.672° E (Fig. 1). The lake covers an approximate surface area of 1.04 km^2^ and forms part of an interconnected lake system within the Bangalore metropolitan region. The lake receives inflows from upstream water bodies and surrounding catchments, with significant contributions from domestic sewage, industrial effluents, and urban stormwater runoff. The region experiences a tropical savanna climate (Köppen classification Aw) characterised by a dry summer period from March to May, the southwest monsoon from June to September, and a post-monsoon period extending from October to February. Mean annual rainfall in the region is approximately 970 mm, with the majority occurring during the monsoon months. The lake is a shallow, artificial water body impounded for irrigation purposes, with depths typically ranging from 1 to 3 m. No systematic in situ monitoring programme was in operation during the study period.

### Satellite data acquisition and preprocessing

Sentinel-2 Level-2A Surface Reflectance imagery was acquired through Google Earth Engine for the period January 2023 to December 2025. The harmonised Sentinel-2 Level-2A product (COPERNICUS/S2_SR_HARMONIZED) was utilised, incorporating atmospheric correction using the Sen2Cor processor providing bottom-of-atmosphere reflectance values (Main-Knorn et al. 2017). Cloud masking was performed using the Sentinel-2 Cloud Probability dataset and Scene Classification Layer. Pixels with cloud probability exceeding 40% were masked, along with cloud shadow (class 3), medium cloud (class 8), high cloud (class 9), and cirrus (class 10) pixels. Images with overall cloudy pixel percentage exceeding 50% were excluded. A total of 100 Sentinel-2 images meeting quality criteria were identified, yielding 93 valid observations for water quality parameter retrieval across 28 months. Monsoon months (June to September) were substantially affected by cloud cover and contributed limited valid observations. Analysis was confined to the complete years 2023–2025 to ensure balanced annual comparisons; the year 2025 ended with 38 valid observations while 2023 and 2024 contributed 30 and 28 respectively. The difference between the 107 images initially identified and the 100 retained for analysis reflects removal of scenes with insufficient water pixel coverage after cloud masking.

### Water quality parameter retrieval algorithms

Six water quality parameters were derived from Sentinel-2 surface reflectance data using established empirical and semi-analytical algorithms. Surface reflectance values were obtained by multiplying digital numbers by a scaling factor of 0.0001. All algorithms were developed and validated for other water bodies; in the absence of site-specific in situ calibration data for Bellandur Lake, the derived concentrations should be interpreted as indicative of relative spatial and temporal patterns rather than precise absolute values (Odermatt et al. 2012; Greb et al. 2018). These six parameters were selected because they are the optically active water quality constituents (chlorophyll-a, turbidity, TSS, CDOM, Secchi depth, and floating algae) for which established empirical or semi-analytical band-ratio algorithms exist and can be applied without a locally calibrated training dataset. A substantially larger set of water quality variables has been explored in the wider Sentinel-2 literature, including dissolved oxygen, total nitrogen, total phosphorus, biochemical/chemical oxygen demand, and pH; however, these are optically non-active constituents that lack a direct radiative-transfer relationship with surface reflectance and instead require machine-learning models calibrated against concurrent in situ measurements, typically yielding weaker and more site-specific retrieval accuracy than the optically active parameters addressed here (Espinoza et al. 2025; Adjovu et al. 2025). No in situ dataset was available for Bellandur Lake to support such calibration; the present study is therefore deliberately restricted to the six optically active parameters for which literature-validated empirical algorithms could be applied without local recalibration.

#### Chlorophyll-a concentration

Chlorophyll-a concentration was estimated using two methods. The Normalised Difference Chlorophyll Index (NDCI) model following Mishra and Mishra (2012) was designated as the primary method, given its stronger theoretical basis for highly turbid and eutrophic conditions and its widely documented performance in hypereutrophic inland waters (Matthews and Bernard 2013; Zhang et al. 2012):

1 $$\mathrm{N}\mathrm{D}\mathrm{C}\mathrm{I}=\frac{{R}_{B5}-{R}_{B4}}{{R}_{B5}+{R}_{B4}}$$

2 $$\mathrm{C}\mathrm{h}\mathrm{l}-{\mathrm{a}}_{\mathrm{N}\mathrm{D}\mathrm{C}\mathrm{I}}=194.325\times {\mathrm{NDCI}}^{2}+86.115\times \mathrm{N}\mathrm{D}\mathrm{C}\mathrm{I}+14.039$$

A red-edge band ratio model was also computed for methodological comparison:

3 $$\mathrm{C}\mathrm{h}\mathrm{l}-{\mathrm{a}}_{\mathrm{R}\mathrm{E}}=86.115\times \frac{{R}_{B5}}{{R}_{B4}}+14.039$$

where *R* represents surface reflectance in the respective spectral bands (B4: 665 nm, B5: 705 nm). All subsequent trend analysis, trophic state calculations, compliance assessments, and spatial analyses utilise the NDCI-based estimates as the primary chlorophyll-a variable.

#### Turbidity

Turbidity in formazin nephelometric units was estimated using the single-band algorithm developed by Dogliotti et al. (2015), validated across a range of 1 to 1000 FNU with mean absolute percentage errors below 30%:

4 $${\mathrm{Turbidity}}_{\mathrm{F}\mathrm{N}\mathrm{U}}=\frac{228.1\times {R}_{B4}}{1-\frac{{R}_{B4}}{0.1641}}$$

This algorithm has been validated for turbidity values ranging from 1 to 1000 FNU and is suitable for moderately to highly turbid inland waters. Formazin nephelometric units (FNU) and nephelometric turbidity units (NTU) both quantify light scattered by suspended particles at a 90° detection angle relative to the incident beam and are both calibrated against a formazin polymer reference standard, but they are not defined identically: FNU denotes measurement under the ISO 7027 protocol using a near-infrared (860 nm) light source, whereas NTU denotes measurement under the US EPA Method 180.1 protocol using a white light source. Although the two scales are frequently treated as numerically interchangeable, instruments compliant with the same ISO 7027 design standard have been shown to vary approximately twofold in absolute response to identical suspensions, so FNU values should not be assumed strictly equivalent to NTU values without site-specific cross-calibration (Kitchener et al. 2017; Davies-Colley et al. 2021). FNU is reported throughout this study because it corresponds to the calibration basis of the Dogliotti et al. (2015) algorithm used here.

#### Total suspended solids

Total suspended solids concentration in mg/L were estimated using an empirical relationship with red band reflectance (Nechad et al. 2010):

5 $$\mathrm{T}\mathrm{S}\mathrm{S}=3957.0\times {R}_{B4}+13.0$$

It is noted that both turbidity and TSS are derived from the Sentinel-2 red band (B4), which partially explains the strong correlation observed between these parameters (*r* = 0.852). Single-band red algorithms of this type are known to perform well in moderately turbid coastal systems but exhibit reduced accuracy under extreme conditions where multiple optically active constituents co-dominate the spectral signal (Nechad et al. 2010; Pahlevan et al. 2022). In waters characterised by the simultaneous presence of high phytoplankton biomass, high inorganic suspended matter, elevated CDOM, and surface organic films precisely the conditions documented at Bellandur Lake, the B4 reflectance signal becomes a composite of contributions from all these constituents rather than a pure sediment proxy; and single-constituent linear algorithms are expected to introduce substantial uncertainty (Dogliotti et al. 2015; Jaywant and Arif 2024). Specifically, chlorophyll-a absorption in the red band can suppress reflectance at 665 nm relative to a purely inorganic TSS signal, while surface foam and floating algal accumulations can artificially elevate or depress B4 reflectance depending on their optical thickness (Hu 2022). The high TSS values observed (mean 221.30 mg/L) alongside moderate Secchi depths (~ 2.27 m) are therefore inconsistent with purely TSS-driven optical models, strongly suggesting that the algorithm overestimates true inorganic suspended matter in this optically complex environment. This does not invalidate the TSS time series as an indicator of relative particulate loading variability the decreasing trend in TSS remains a meaningful finding but the absolute concentrations should be interpreted with caution and should not be compared directly with gravimetrically determined TSS values from other studies.

#### Coloured dissolved organic matter

Coloured dissolved organic matter, also referred to as chromophoric dissolved organic matter or gelbstoff, is the optically active fraction of dissolved organic matter that absorbs light strongly in the ultraviolet and blue-visible spectral region, with absorption decreasing approximately exponentially with increasing wavelength toward the red and near-infrared; this well-established spectral behaviour is the physical basis for empirical retrieval algorithms built on blue-to-green reflectance ratios, including the one applied here (Brezonik et al. 2015; Marinho et al. 2024). Compositionally, CDOM is dominated by humic and fulvic acids together with lower-molecular-weight aromatic amino acids and lignin-derived phenolic compounds, sourced from both allochthonous inputs (soil and vegetation leachate, sewage, and industrial effluent) and autochthonous production (microbial and algal decomposition products) (Marinho et al. 2024). In sewage-impacted urban systems such as Bellandur Lake, CDOM is expected to be dominated by anthropogenic inputs of partially treated wastewater and decaying organic matter rather than by purely terrestrial humic sources, consistent with the elevated CDOM values reported here and with the lake’s documented sewage and industrial effluent loading (Das et al. 2023; Ramachandra and Kumar 2008).

The coloured dissolved organic matter index was derived using the blue-to-green band ratio following Brezonik et al. (2015):

6 $$\mathrm{C}\mathrm{D}\mathrm{O}\mathrm{M}=537.0\times {e}^{-2.93\times \frac{{R}_{B2}}{{R}_{B3}}}$$

where R_B2 and R_B3 represent reflectance in the blue (490 nm) and green (560 nm) bands respectively.

#### Secchi disk depth

Water transparency expressed as Secchi disk depth in metres was estimated using the relationship developed by Binding et al. (2007):

7 $${\mathrm{Secchi}}_{\mathrm{D}\mathrm{e}\mathrm{p}\mathrm{t}\mathrm{h}}=-1.22\times \mathrm{l}\mathrm{n}\left(\frac{{R}_{B2}}{{R}_{B3}}\right)+1.65$$

Both CDOM and Secchi depth are derived from the ratio of Sentinel-2 blue (B2) and green (B3) bands. The very strong correlation observed between CDOM and Secchi depth (*r* = 0.962) is in part a consequence of this shared spectral input rather than exclusively an environmental relationship, and should be interpreted accordingly. This mathematical coupling between blue-green ratio-based algorithms is a well-documented artefact in remote sensing of inland waters (Huang et al. 2025; Brezonik et al. 2015). Furthermore, the B2/B3 ratio is sensitive to thin haze, adjacency effects from surrounding urban surfaces, and the optical influence of dissolved organic compounds common in sewage-impacted systems, all of which can introduce uncertainty in both CDOM and Secchi depth retrievals independently of true water quality variation (Odermatt et al. 2012; Salls et al. 2024). The positive correlation between chlorophyll-a and Secchi depth observed in this study (*r* = 0.854) is counterintuitive and is likely attributable to CDOM dominating light attenuation rather than phytoplankton biomass; Secchi depth therefore does not reliably reflect trophic state in this system.

#### Floating algae index

The floating algae index was calculated following Hu (2009) to detect surface algal accumulations and floating vegetation:

8 $$\mathrm{F}\mathrm{A}\mathrm{I}={R}_{B8}-\left[{R}_{B4}+({R}_{B11}-{R}_{B4})\times \frac{842-665}{1610-665}\right]$$

where R_B8, R_B4, and R_B11 represent reflectance in the near infrared (842 nm), red (665 nm), and shortwave infrared (1610 nm) bands respectively. Positive FAI values indicate the presence of floating algae or aquatic vegetation.

### Temporal aggregation and statistical analysis

Individual scene observations were aggregated to monthly composites using median values to reduce noise. Spatial averaging generated lake-averaged time series for temporal trend analysis. Seasonal analyses were based on four meteorological seasons: December–February (DJF), March–May (MAM), June–August (JJA), and September–November (SON). Dispersion is reported using the interquartile range (IQR) rather than standard deviation, consistent with the use of median as the measure of central tendency.

### Trend analysis

Temporal trends were assessed using the Mann–Kendall nonparametric test (Mann 1945; Kendall 1975), which is robust against non-normality and suitable for detecting monotonic trends. The Mann–Kendall statistic *S* is computed as follows:

9 $$S={\sum}_{i=1}^{n-1}{\sum}_{j=i+1}^{n}{\mathrm{sgn}}({x}_{j}-{x}_{i})$$

where sgn(θ) = + 1 if θ > 0, 0 if θ = 0, and − 1 if θ < 0. A positive *S* indicates an increasing trend. The normalised Kendall tau (*τ*) is as follows:

10 $$\tau =\frac{S}{n(n-1)/2}$$

Trend magnitude was quantified using Sen’s slope estimator (Sen 1968):

11 $$Q={\mathrm{median}}\left[\frac{{x}_{j}-{x}_{i}}{j-i}\right]\text{ for all }j>i$$

where *Q* is the median slope across all data pairs, providing a robust estimate of the rate of change per observation. For pixel-wise trend analysis, the Benjamini–Hochberg false discovery rate (FDR) correction was applied to the *p* values from all 8829 pixels tested simultaneously, controlling the expected proportion of false positives at *q* < 0.05 (Benjamini and Hochberg 1995). Under this procedure, *p* values are rank-ordered *p*₁ ≤ *p*₂ ≤ … ≤ *pₘ*, and the largest *k* is found such that *pₖ* ≤ (*k*/*m*)*α*, where *m* is the total number of tests and *α* = 0.05. This correction is essential given the large number of simultaneous statistical tests and the spatial autocorrelation inherent in adjacent pixels originating from shared satellite scenes. It is acknowledged that neighbouring pixels are not fully independent observations due to shared atmospheric and illumination conditions; the FDR-corrected results should therefore be interpreted as indicative of broad spatial patterns rather than independently confirmed pixel-level trends.

### Seasonal comparison

Pre-monsoon (March, April, May) and post-monsoon (October, November) water quality conditions were compared using the Mann–Whitney *U* test (Mann and Whitney 1947). For two independent samples of size *n*₁ and *n*₂, the test statistic *U* is as follows:

12 $$U={n}_{1}{n}_{2}+\frac{{n}_{1}({n}_{1}+1)}{2}-{R}_{1}$$

where *R*₁ is the sum of ranks of the first sample in the combined ranking. The smaller of *U* and (*n*₁*n*₂ − *U*) is used for significance testing. The test is equivalent to testing whether the probability that a randomly selected observation from the pre-monsoon period exceeds a randomly selected post-monsoon observation is equal to 0.5. This test was selected because it makes no distributional assumptions and is appropriate for the moderate sample sizes available. Percentage change in median values between periods was calculated for each parameter.

### Trophic state assessment

Trophic status was evaluated using the Carlson trophic state index (Carlson 1977). Given that Secchi depth in Bellandur Lake is strongly influenced by CDOM rather than phytoplankton biomass (evidenced by CDOM–Secchi *r* = 0.962 and the counterintuitive positive chlorophyll–Secchi relationship), TSI(Secchi) does not reliably reflect trophic conditions in this system. Accordingly, trophic classification is based exclusively on TSI(Chl-a) derived from the NDCI-based chlorophyll-a estimates:

13 $$\mathrm{TSI(Chl-a)}=9.81\times \mathrm{ln}\left({\mathrm{Chl-a}}_{\mathrm{NDCI}}\right)+30.6$$

TSI(Secchi) is reported for diagnostic comparison only and is not used for trophic classification. The trophic thresholds are as follows: oligotrophic (TSI < 30), mesotrophic (30 ≤ TSI < 50), eutrophic (50 ≤ TSI < 70), and hypereutrophic (TSI ≥ 70).

### Water quality standards compliance

Compliance was assessed against Central Pollution Control Board of India standards for total suspended solids (class D standard for fish and wildlife propagation: 100 mg/L) and WHO/OECD chlorophyll-a trophic threshold. Given the absence of in situ validation, these comparisons are presented as indicative assessments of relative pollution severity rather than formal regulatory determinations.

### Water quality index

A composite water quality index (WQI) was calculated by normalising parameters to a 0–100 scale and applying empirical weights:

14 $${\mathrm{WQI}}=0.30\times (100-{\mathrm{Chl-a}}_{\mathrm{norm}})+0.25\times (100-{\mathrm{Turb}}_{\mathrm{norm}})+0.25\times (100-{\mathrm{TSS}}_{\mathrm{norm}})+0.20\times {\mathrm{SD}}_{\mathrm{norm}}$$

where ‘norm’ denotes min–max normalisation of each parameter to its 2nd–98th percentile range across the full study dataset:

15 $${X}_{\mathrm{norm}}=\frac{X-{X}_{p2}}{{X}_{p98}-{X}_{p2}}\times 100$$

with *X*_*p*2_ and *X*_*p*98_ representing the 2nd and 98th percentile values respectively. Higher WQI values indicate relatively better water quality within the 2023–2025 study period. Weights reflect the relative ecological importance assigned to algal biomass (0.30), particulate loading via turbidity and TSS (0.25 each), and water transparency (0.20). The WQI is an internally normalised relative index and should not be compared directly with indices calibrated against in situ measurements.

### Software and data processing

Satellite data processing was conducted using Google Earth Engine API in Python environment (Gorelick et al. 2017). Statistical analyses were performed using Python with scipy and pymannkendall libraries. Visualisations were generated using matplotlib and seaborn packages.

## Results

### Baseline water quality conditions

Analysis of 93 valid observations across January 2023 to December 2025 revealed severely degraded water quality conditions in Bellandur Lake. NDCI-based chlorophyll-a concentrations ranged from 18.23 to 119.37 µg/L, with a mean of 71.31 µg/L and median of 70.24 µg/L (Table 1). The coefficient of variation was 30.5%, indicating moderate temporal variability. For methodological comparison, the red-edge ratio model yielded substantially higher values (mean 210.28 µg/L, median 201.56 µg/L, CV 26.2%). Turbidity exhibited the highest variability among all parameters (CV = 94.7%), ranging from 6.91 to 187.32 FNU with a mean of 41.67 FNU and median of 24.86 FNU. Total suspended solids ranged from 105.84 to 470.95 mg/L with a mean of 221.30 mg/L. CDOM values spanned 17.52 to 398.97 (CV = 59.1%). Secchi depth demonstrated the lowest variability (CV = 16.9%), ranging from 1.45 to 3.53 m with a mean of 2.27 m. The floating algae index maintained consistently positive values throughout the study period (mean 0.16, range 0.01 to 0.28).

**Table 1:** Descriptive statistics of water quality parameters for Bellandur Lake (January 2023 to December 2025, *n* = 93). Values report mean and median. The red-edge ratio estimate is retained for methodological comparison

Parameter	Unit	Mean	SD	Min	25th	Median	75th	Max	CV (%)
Chlorophyll-a (NDCI)	µg/L	71.31	21.78	18.23	57.34	70.24	84.70	119.37	30.5
Chlorophyll-a (red-edge)	µg/L	210.28	55.10	107.57	176.07	201.56	231.71	422.69	26.2
Turbidity	FNU	41.67	39.48	6.91	14.00	24.86	64.87	187.32	94.7
Total suspended solids	mg/L	221.30	83.76	105.84	160.02	200.92	263.04	470.95	37.8
CDOM	index	101.02	59.71	17.52	64.44	86.86	111.08	398.97	59.1
Secchi depth	m	2.27	0.38	1.45	2.04	2.20	2.39	3.53	16.9
Floating algae index	—	0.16	0.07	0.01	0.11	0.18	0.22	0.28	40.5

*SD* standard deviation, *CV* coefficient of variation, *CDOM* coloured dissolved organic matter, *FNU* formazin nephelometric units, *RE* red-edge ratio model. All values are indicative given the absence of site-specific in situ validation

### Temporal trends

**Fig. 2:**
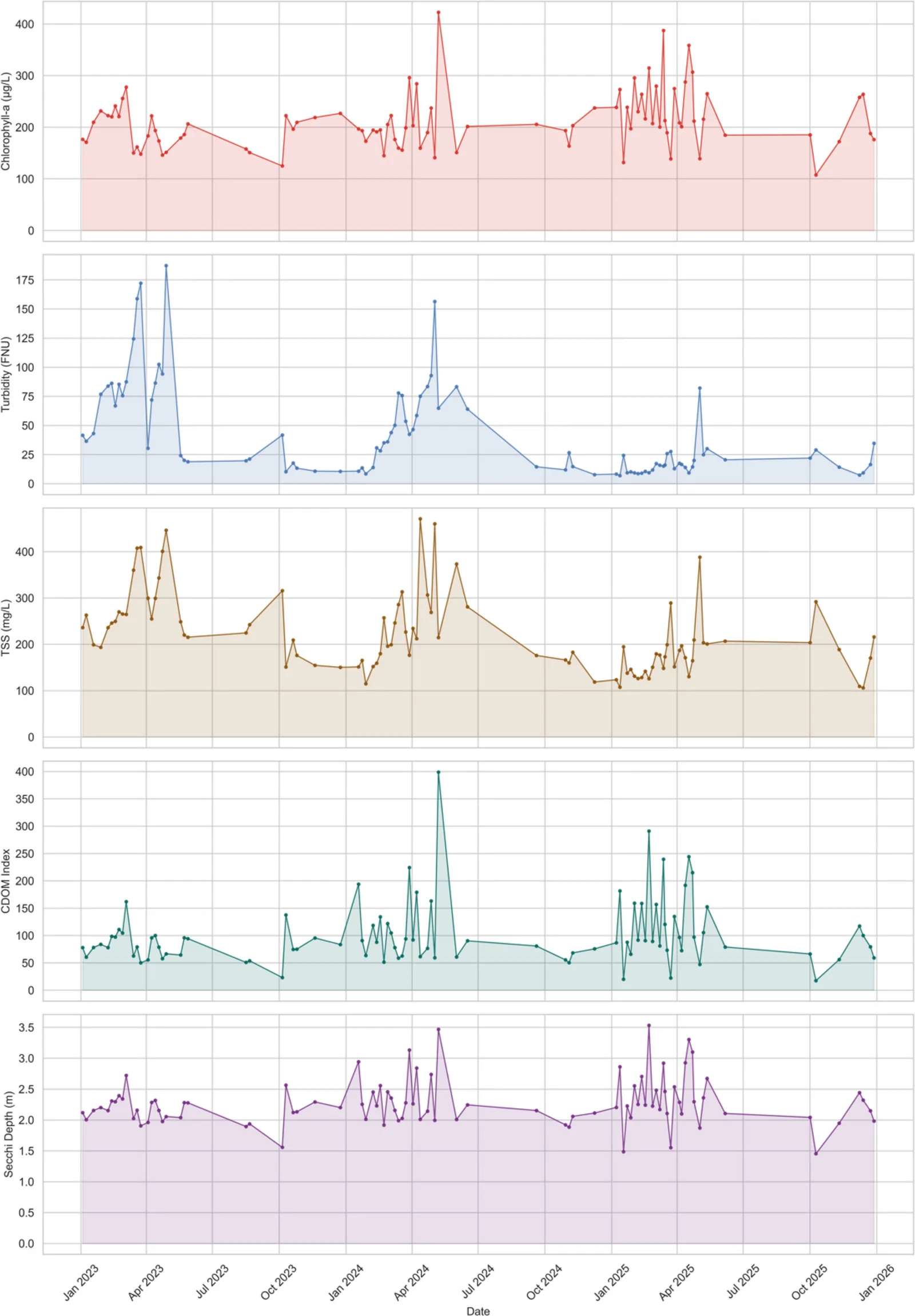
Temporal variation of water quality parameters in Bellandur Lake from January 2023 to December 2025. **a** Chlorophyll-a. **b** Turbidity. **c** Total suspended solids. **d** Coloured dissolved organic matter. **e** Secchi depth. Note: the statistically significant Mann–Kendall trend identified for chlorophyll-a (panel **a**; Table 2) is a weak monotonic signal (*τ* = 0.144) superimposed on substantial short-term variability and is not necessarily apparent from visual inspection of panel (**a**) alone; see “Temporal trends” section for discussion

Mann–Kendall trend analysis identified statistically significant monotonic trends for four of the six water quality parameters examined (Table 2). NDCI-based chlorophyll-a exhibited a significant increasing trend (*τ* = 0.144, *p* = 0.041, Sen’s slope = 0.188 µg/L per observation), a finding not apparent when using the red-edge ratio model (*τ* = 0.129, *p* = 0.068). It is emphasised that *τ* = 0.144 corresponds to a weak monotonic association, and the Sen’s slope (0.188 µg/L per observation, equivalent to approximately 5.3 µg/L over the 28-month record) is small relative to the substantial background variability of NDCI chlorophyll-a (CV = 30.5%, Table 1). Consequently, this statistically significant trend is not expected to be, and is not, readily discernible by eye in the raw noisy time series shown in Fig. 2a; its detection instead reflects the rank-based sensitivity of the Mann–Kendall test to a persistent monotonic tendency rather than a large or visually obvious directional shift. Turbidity exhibited a highly significant decreasing trend (*p* < 0.001) with a Kendall tau of − 0.298 and Sen’s slope of − 0.407 FNU per observation. Total suspended solids demonstrated a significant decreasing trend (*p* = 0.001) with a Kendall tau of − 0.268 and Sen’s slope of − 1.032 mg/L per observation. The floating algae index showed a strongly significant increasing trend (*τ* = 0.419, *p* < 0.001) with a Sen’s slope of 0.002 per observation, representing the strongest trend among all parameters. Neither CDOM (*p* = 0.293) nor Secchi depth (*p* = 0.368) exhibited significant trends. The temporal evolution of parameters is illustrated in Fig. 2.

**Table 2:** Mann–Kendall trend analysis results for lake-averaged water quality parameters (January 2023 to December 2025, *n* = 93). NDCI-based chlorophyll-a is used as the primary variable

Parameter	Trend	*τ*	*S*	Sen’s slope	*p* value	Significance
Chlorophyll-a (NDCI)	Increasing	0.144	616	0.188 µg/L per obs	0.041	*
Turbidity	Decreasing	− 0.298	− 1276	− 0.407 FNU per obs	< 0.001	*
Total suspended solids	Decreasing	− 0.268	− 1148	− 1.032 mg/L per obs	0.001	*
CDOM	No trend	0.074	318	0.156 per obs	0.293	ns
Secchi depth	No trend	0.064	272	0.001 m per obs	0.368	ns
Floating algae index	Increasing	0.419	1792	0.002 per obs	< 0.001	*

*Significant at* α* = 0.05;* ns *not significant

### Annual variations

**Fig. 3:**
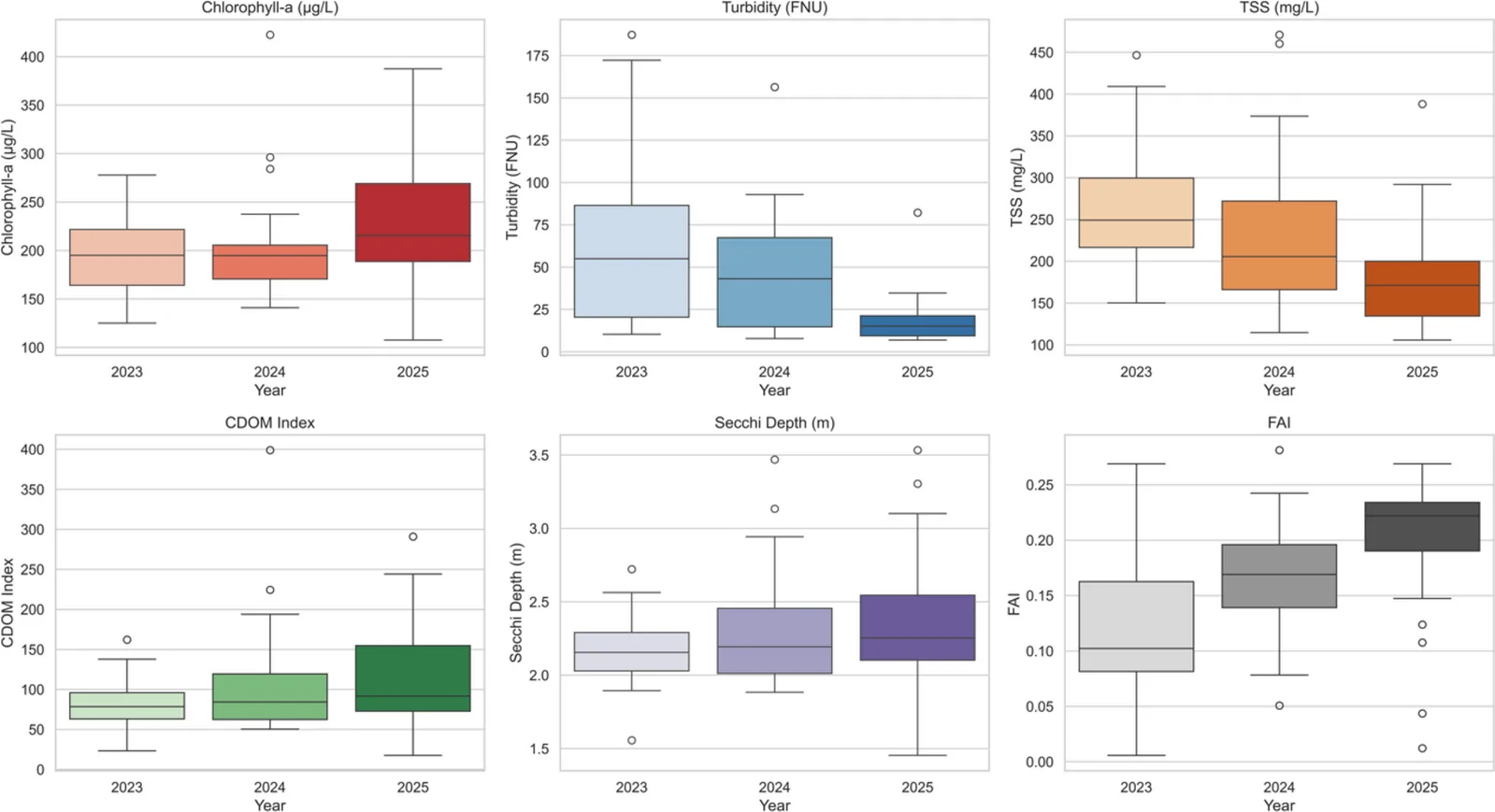
Annual comparison of water quality parameters in Bellandur Lake (2023–2025). Box plots show median and interquartile range

Annual median water quality parameters for the three complete study years (2023–2025) revealed distinct temporal patterns (Table 3). NDCI-based chlorophyll-a concentrations remained stable between 2023 (67.57 µg/L [IQR 28.48]) and 2024 (67.37 µg/L [IQR 17.30]) before increasing to 78.21 µg/L (IQR 33.84) in 2025, a cumulative increase of 15.9%. Turbidity decreased progressively from 54.98 FNU in 2023 to 15.13 FNU in 2025, a 72.5% reduction. Total suspended solids followed a similar declining trajectory, decreasing from 249.22 to 171.17 mg/L, a 31.3% reduction. The floating algae index exhibited the largest relative change, rising from 0.10 in 2023 to 0.22 in 2025, a 117.0% increase. CDOM increased from 78.42 to 91.74 (+ 17.0%), and Secchi depth improved modestly from 2.16 to 2.25 m (+ 4.6%). Annual variations across all parameters are illustrated in Fig. 3.

**Table 3:** Annual median values [IQR] and percentage changes in water quality parameters (2023–2025)

Parameter	Unit	2023 (*n* = 30)	2024 (*n* = 28)	2025 (*n* = 35)	2023 → 2025
Chlorophyll-a (NDCI)	µg/L	67.57 [IQR 28.48]	67.37 [IQR 17.30]	78.21 [IQR 33.84]	+ 15.9%
Turbidity	FNU	54.98 [IQR 66.00]	43.15 [IQR 52.74]	15.13 [IQR 11.85]	− 72.5%
TSS	mg/L	249.22 [IQR 82.96]	205.66 [IQR 105.96]	171.17 [IQR 65.46]	− 31.3%
CDOM	index	78.42 [IQR 32.77]	84.36 [IQR 57.07]	91.74 [IQR 81.85]	+ 17.0%
Secchi depth	m	2.16 [IQR 0.26]	2.19 [IQR 0.44]	2.25 [IQR 0.44]	+ 4.6%
FAI	—	0.10 [IQR 0.08]	0.17 [IQR 0.06]	0.22 [IQR 0.04]	+ 117.0%

### Seasonal patterns

**Fig. 4:**
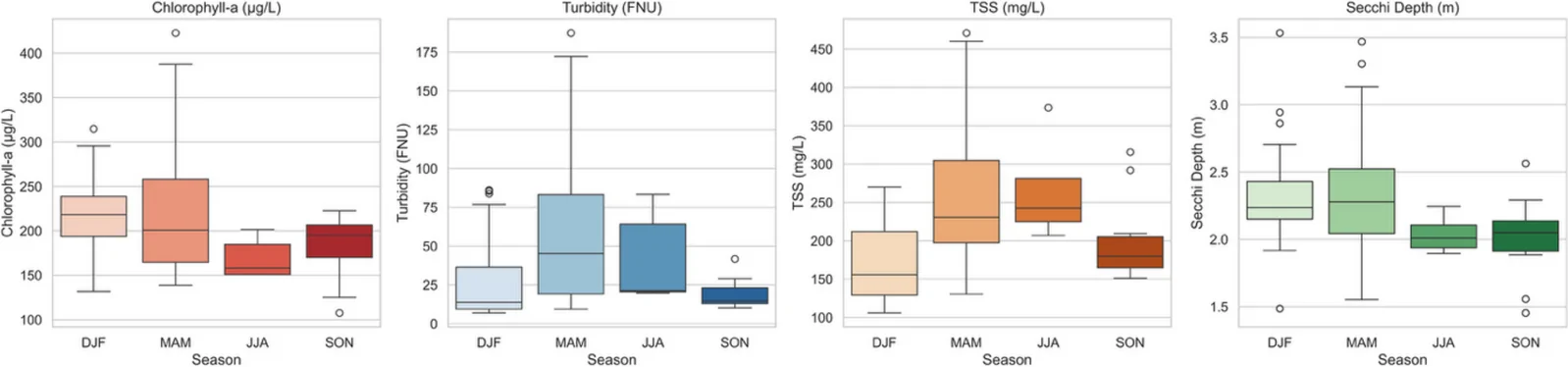
Seasonal variation in water quality parameters across meteorological seasons: DJF (December–February), MAM (March–May), JJA (June–August), and SON (September–November). Box plots show median and interquartile range

Seasonal analysis revealed pronounced differences in water quality parameters across meteorological seasons (Table 4). Pre-monsoon months (MAM) exhibited the highest median turbidity (45.18 FNU [IQR 63.98]) and total suspended solids (230.59 mg/L [IQR 107.33]) among all seasons, coinciding with elevated dust deposition and reduced water levels characteristic of the hot dry season. Post-monsoon months (SON) recorded the lowest turbidity (14.66 FNU [IQR 10.16]) and relatively low TSS (179.61 mg/L [IQR 40.39]). Winter months (DJF) displayed the highest median NDCI chlorophyll-a (77.60 µg/L [IQR 21.02]). Monsoon adjacent observations (JJA, *n* = 5) showed the lowest chlorophyll-a (47.24 µg/L), though this season was represented by only five observations and results should be interpreted cautiously. Seasonal variations are further illustrated in Fig. 4.

**Table 4:** Seasonal statistics (median [IQR]) of water quality parameters. *DJF*, December–February; *MAM*, March–May; *JJA*, June–August (*n* = 5, interpret with caution); *SON*, September–November

Season	Period	*n*	Chl-a NDCI (µg/L)	Turbidity (FNU)	TSS (mg/L)	Secchi depth (m)	FAI
DJF	December to February	34	77.60 [IQR 21.02]	13.78 [IQR 27.04]	155.71 [IQR 82.71]	2.24 [IQR 0.28]	0.14 [IQR 0.11]
MAM	March to May	43	70.32 [IQR 39.78]	45.18 [IQR 63.98]	230.59 [IQR 107.33]	2.28 [IQR 0.48]	0.19 [IQR 0.06]
JJA	June to August	5	47.24 [IQR 17.46]	21.27 [IQR 43.51]	242.40 [IQR 56.38]	2.01 [IQR 0.17]	0.16 [IQR 0.11]
SON	September to November	14	67.42 [IQR 18.74]	14.66 [IQR 10.16]	179.61 [IQR 40.39]	2.05 [IQR 0.23]	0.10 [IQR 0.11]

### Pre monsoon versus post monsoon comparison

**Fig. 5:**
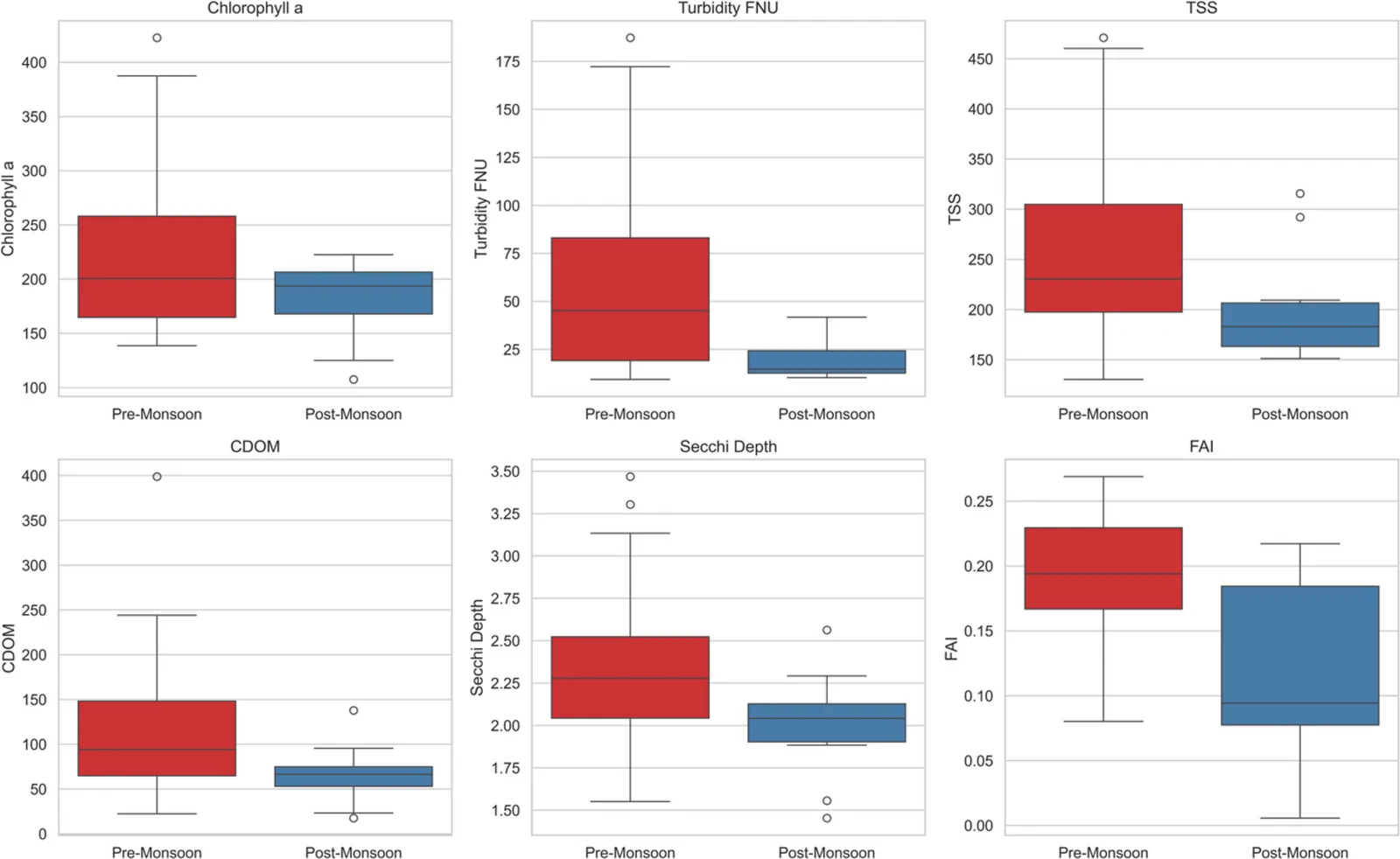
Comparison of water quality parameters between pre-monsoon (March–May, *n* = 42) and post-monsoon (October–November, *n* = 11) periods. Box plots show median and interquartile range

Statistical comparison between pre-monsoon (*n* = 42) and post-monsoon (*n* = 11) periods using the Mann–Whitney* U* test revealed significant differences for five of the six parameters examined (Table 5). Turbidity was significantly lower during post-monsoon periods (14.75 FNU versus 45.18 FNU, − 67.4%, *p* = 0.002). Total suspended solids decreased by 20.6% (*p* = 0.026). CDOM declined by 29.5% (*p* = 0.011) and Secchi depth decreased by 10.4% (*p* = 0.013). The floating algae index showed a 51.4% reduction (*p* = 0.003). NDCI-based chlorophyll-a exhibited a modest 6.8% decrease (70.32 to 65.54 µg/L) that was not statistically significant (*p* = 0.278). Pre-monsoon and post-monsoon distributions are compared in Fig. 5.

**Table 5:** Pre monsoon versus post monsoon comparison using Mann–Whitney *U* test

Parameter	Pre monsoon median (*n* = 42)	Post monsoon median (*n* = 11)	Change (%)	*p* value	Significant
Chlorophyll-a NDCI (µg/L)	70.32	65.54	− 6.8	0.278	No
Turbidity (FNU)	45.18	14.75	− 67.4	0.002	Yes
TSS (mg/L)	230.59	183.01	− 20.6	0.026	Yes
CDOM (index)	94.13	66.34	− 29.5	0.011	Yes
Secchi depth (m)	2.28	2.04	− 10.4	0.013	Yes
FAI	0.19	0.09	− 51.4	0.003	Yes

### Monthly extremes

Monthly extreme values for the study period (2023–2025) are summarised in Table 6. Peak NDCI chlorophyll-a (91.08 µg/L) was recorded in February 2025, while the minimum (40.66 µg/L) occurred in October 2025. Maximum turbidity (141.64 FNU) and total suspended solids (383.92 mg/L) were both observed in March 2023, corresponding to the peak pre-monsoon period. Minimum turbidity (7.85 FNU) and TSS (118.69 mg/L) occurred in December 2024. The highest CDOM (229.16) and Secchi depth (2.73 m) were recorded in May 2024, while their respective minima occurred in October 2025. Peak FAI (0.26) was observed in May 2025.

**Table 6:** Monthly extremes of water quality parameters (January 2023 to December 2025)

Parameter	Unit	Maximum	Month	Minimum	Month
Chlorophyll-a (NDCI)	µg/L	91.08	Feb 2025	40.66	Oct 2025
Turbidity	FNU	141.64	Mar 2023	7.85	Dec 2024
TSS	mg/L	383.92	Mar 2023	118.69	Dec 2024
CDOM	index	229.16	May 2024	41.93	Oct 2025
Secchi depth	m	2.73	May 2024	1.75	Oct 2025
FAI	—	0.26	May 2025	0.07	Jan 2023

**Fig. 6:**
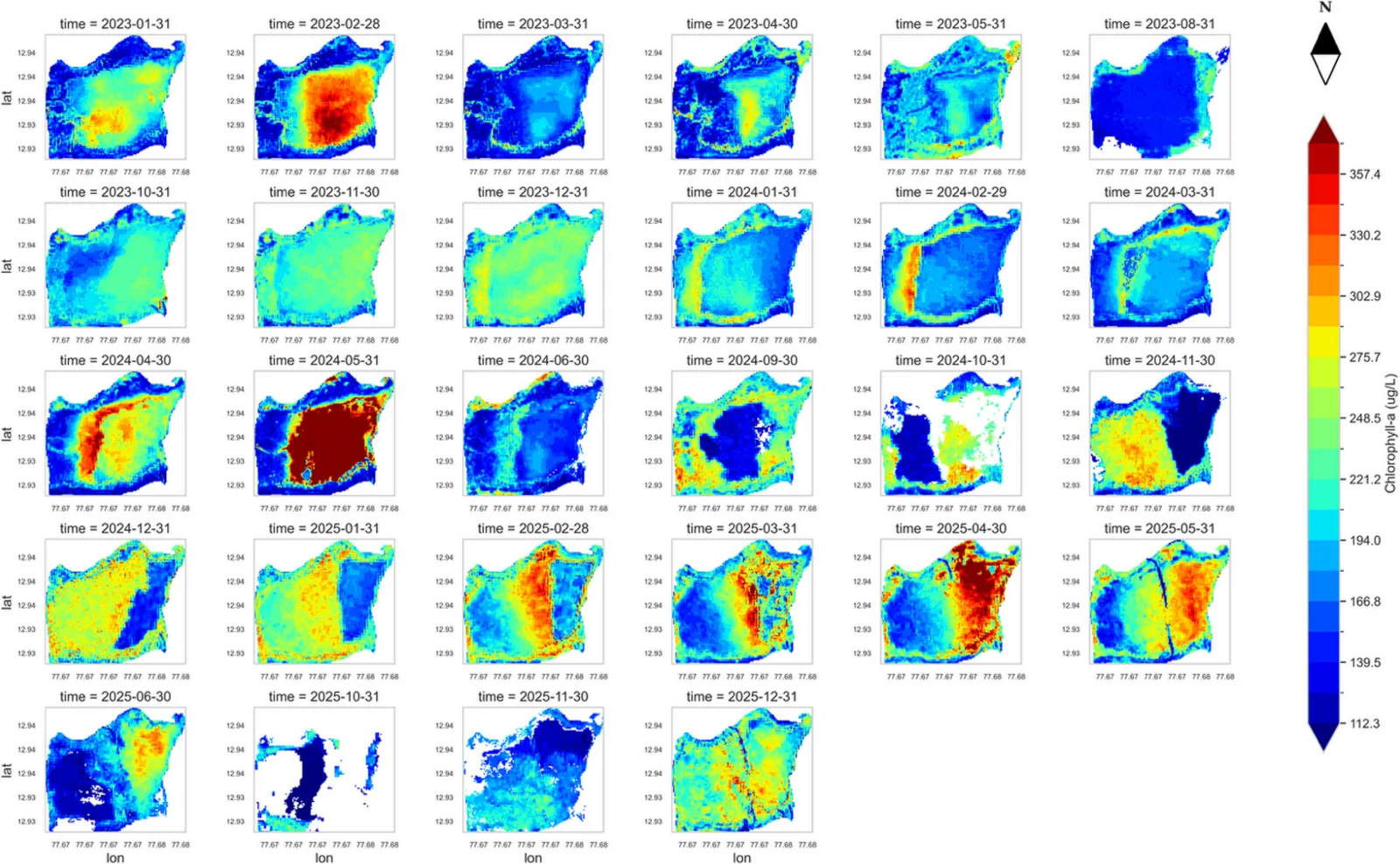
Monthly spatial distribution of chlorophyll-a concentrations (NDCI-based) in Bellandur Lake (January 2023 to December 2025). Colour scale represents NDCI chlorophyll-a in µg/L

**Fig. 7:**
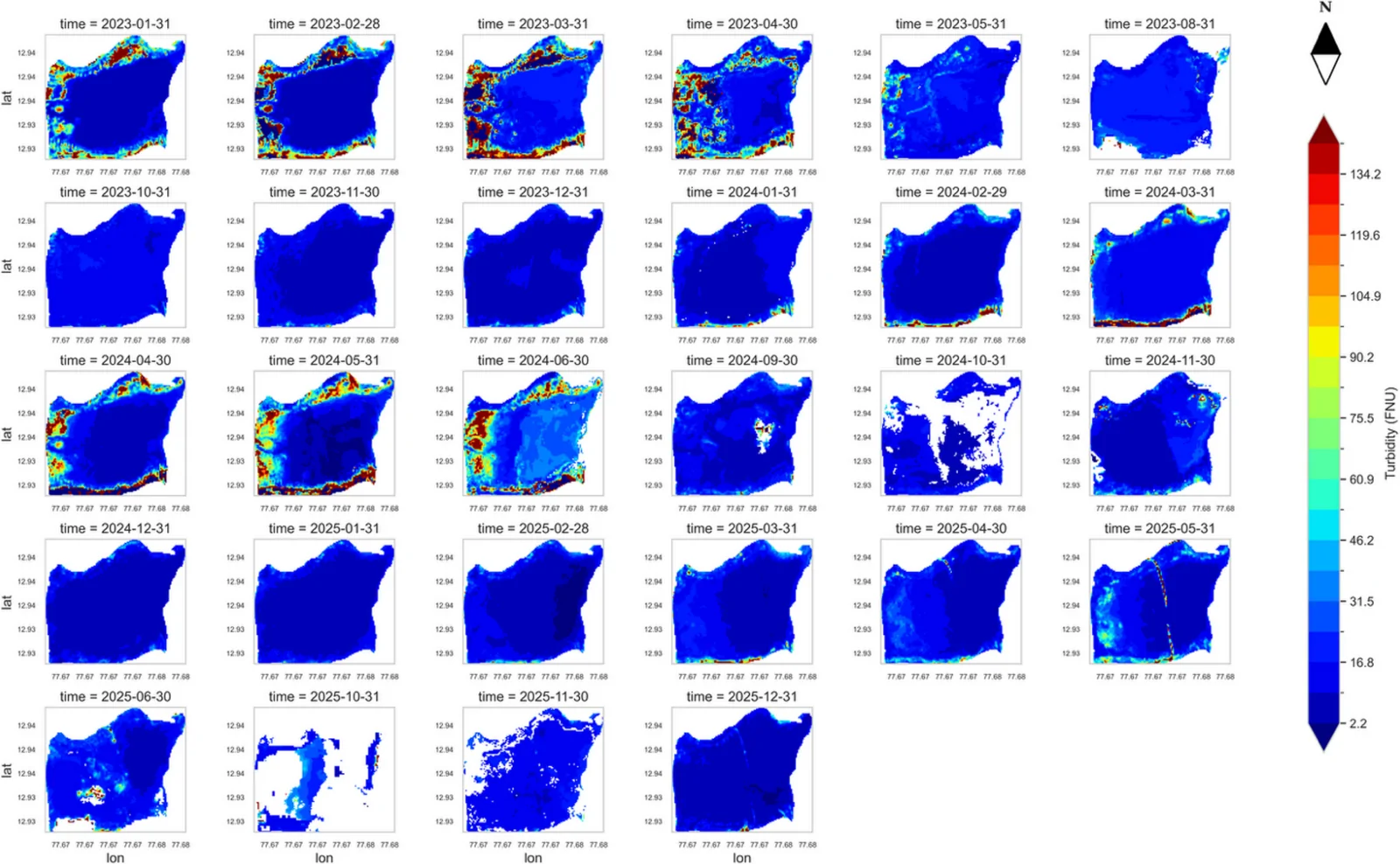
Monthly spatial distribution of turbidity (FNU) in Bellandur Lake (January 2023 to December 2025)

**Fig. 8:**
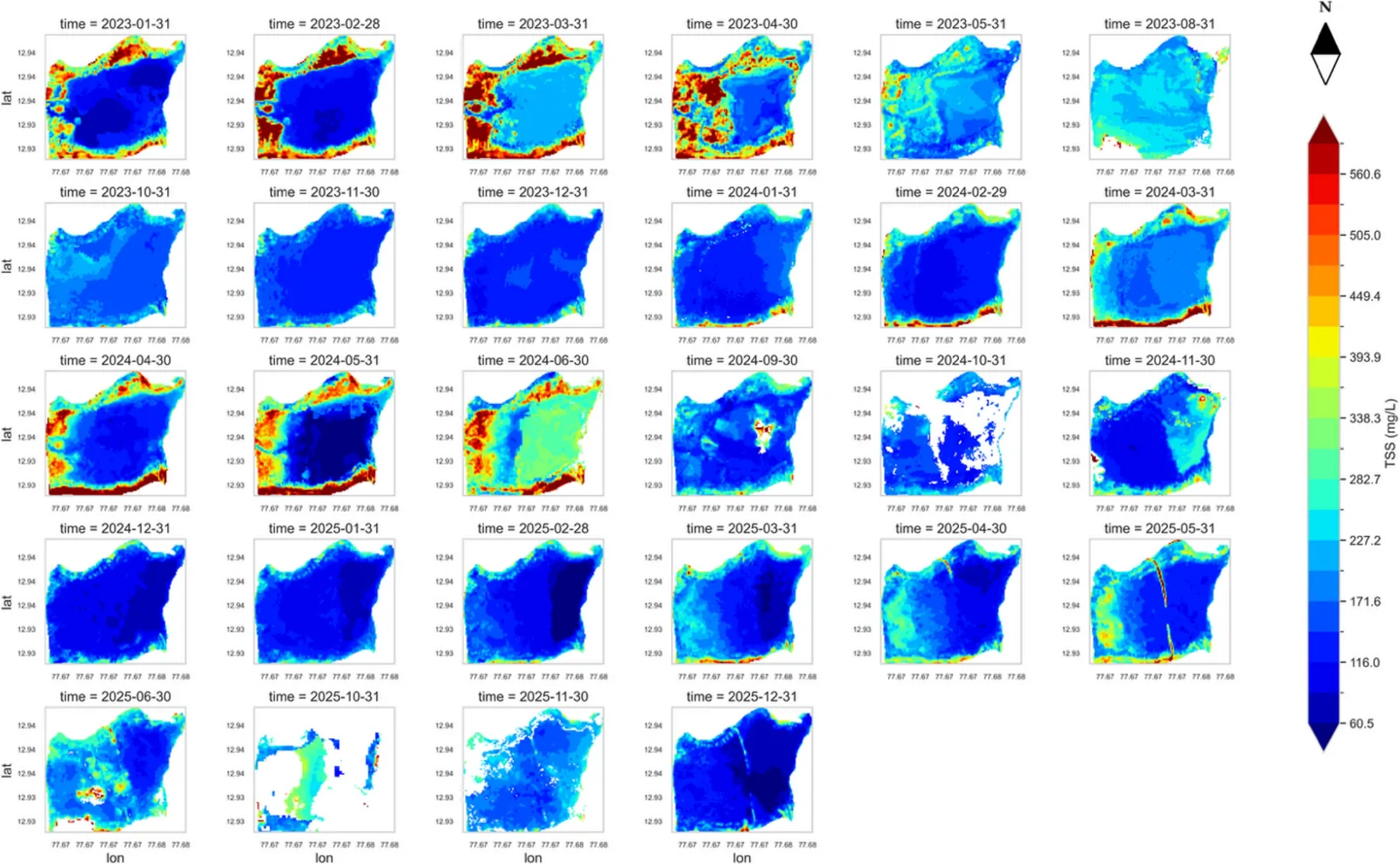
Monthly spatial distribution of total suspended solids (mg/L) in Bellandur Lake (January 2023 to December 2025)

**Fig. 9:**
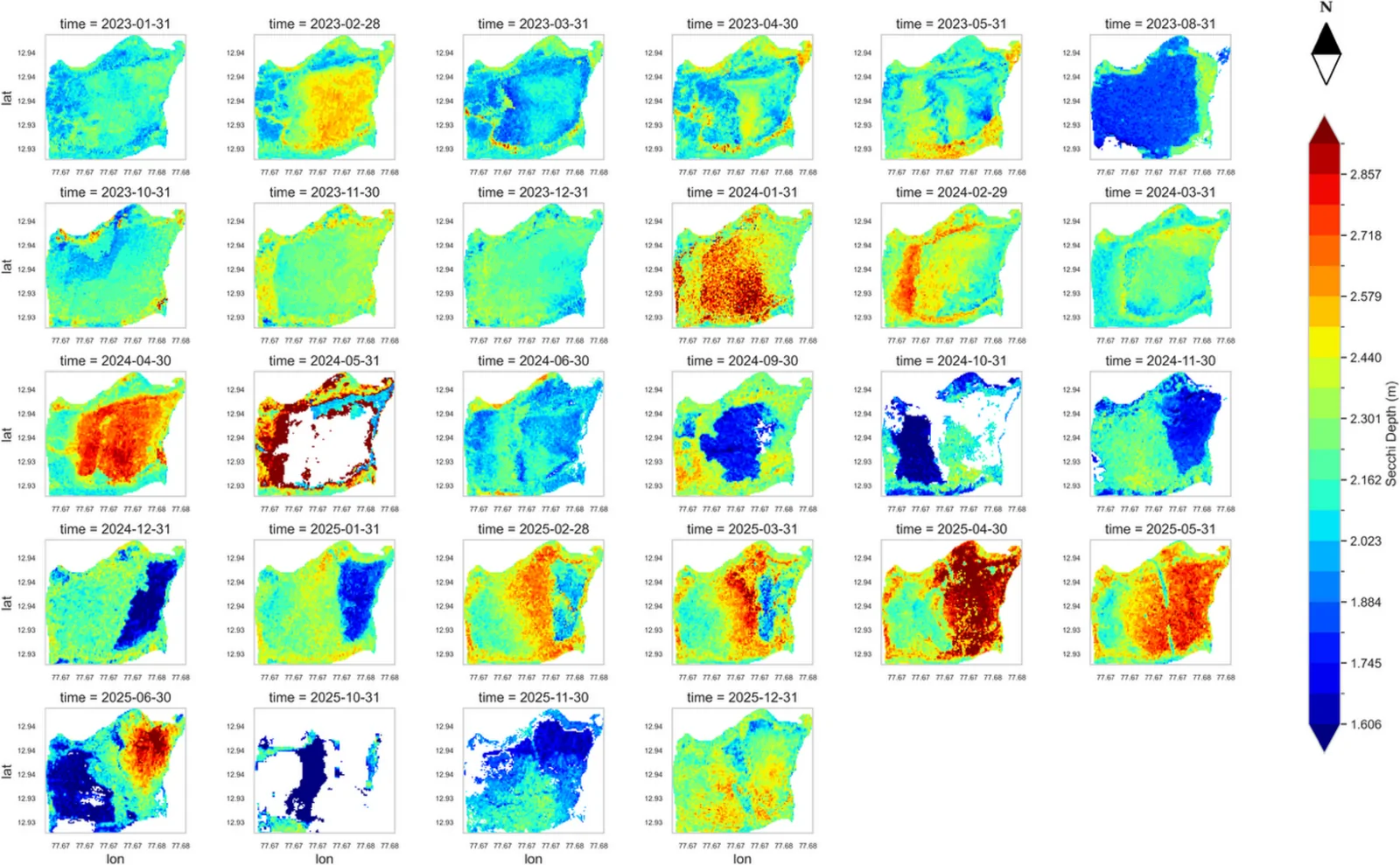
Monthly spatial distribution of Secchi depth (m) in Bellandur Lake (January 2023 to December 2025). Note: The May 2024 panel was inspected for cloud-masking artefacts; any residual masking inconsistencies are discussed in the “Algorithm performance and validation considerations” section

Monthly spatial distribution maps for chlorophyll-a, turbidity, TSS, and Secchi depth (Figs. 6, 7, 8, and 9) are presented in the main text. CDOM and FAI montly maps are provided in the supplementary material (Online Resource 1; Online Resource 2)

#### Trophic state assessment

**Fig. 10:**
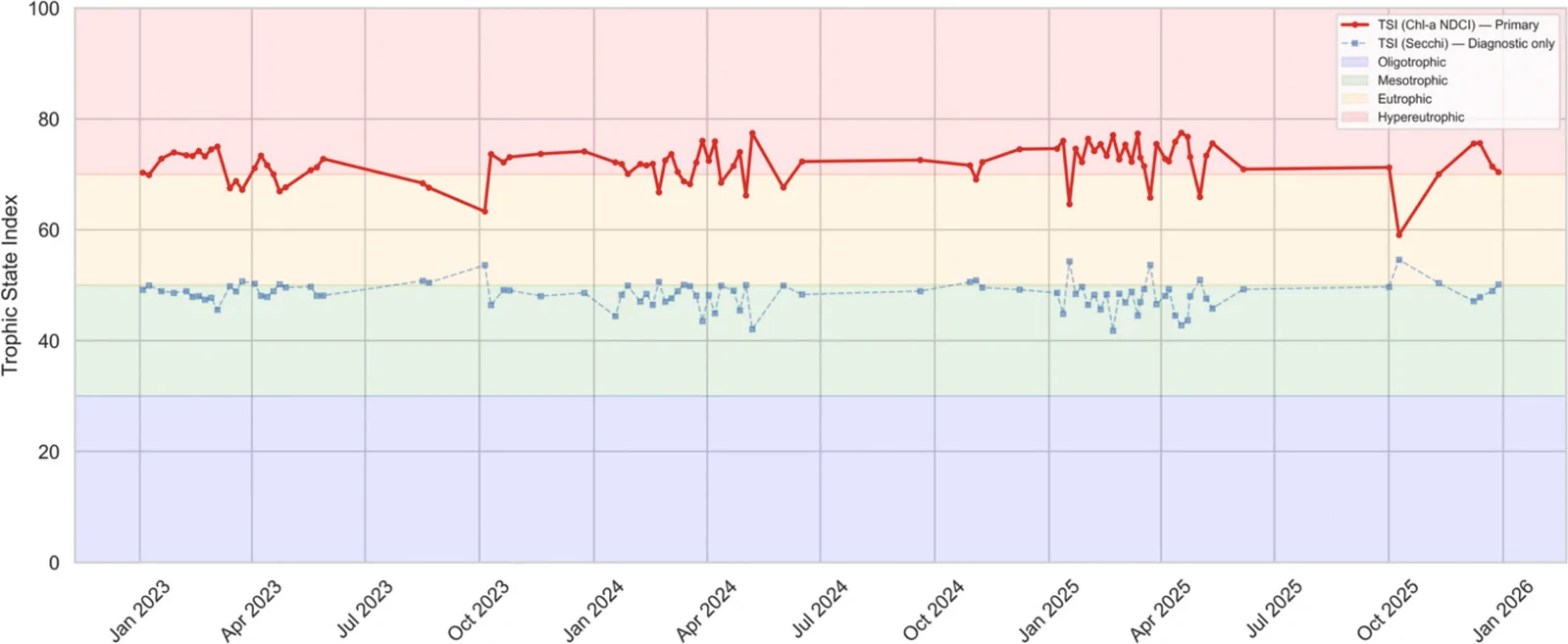
Carlson trophic state index time series for Bellandur Lake (January 2023 to December 2025) showing TSI(Chl-a NDCI) as the primary trophic indicator and TSI(Secchi) for diagnostic comparison. Trophic classification is based on TSI(Chl-a) only. Horizontal bands indicate trophic classification thresholds

Carlson trophic state index calculations based on NDCI chlorophyll-a yielded a mean TSI of 71.94 (range 59.08 to 77.51), consistently exceeding the hypereutrophic threshold of 70. TSI values derived from Secchi depth averaged 48.36 (range 41.81 to 54.61), falling within the mesotrophic to eutrophic range. Under trophic classification based on TSI(Chl-a), 73 of 93 observations (78.5%) were hypereutrophic and 20 (21.5%) were eutrophic. The temporal evolution of TSI values is presented in Fig. 10.

### Water quality standards compliance

Assessment against WHO/OECD chlorophyll-a thresholds using NDCI-based estimates classified 92 of 93 observations (98.9%) as hypereutrophic, with one observation (1.1%) falling in the eutrophic category (Table 7). Total suspended solids exceeded the CPCB class D limit of 100 mg/L in 100% of observations. Water transparency based on Secchi depth showed 17 of 93 observations (18.3%) in the moderate category (1.0 to 2.0 m) and 76 (81.7%) in the good category (> 2.0 m). Compliance results are summarised in Table 7.

**Table 7:** Water quality standards compliance assessment (*n* = 93)

Assessment	Classification	Threshold	Count	Percentage
Trophic status (Chl-a NDCI)	Oligotrophic	< 2.5 µg/L	0/93	0.0%
	Mesotrophic	2.5–8 µg/L	0/93	0.0%
	Eutrophic	8–25 µg/L	1/93	1.1%
	Hypereutrophic	> 25 µg/L	92/93	98.9%
TSS (CPCB class D)	Compliant	≤ 100 mg/L	0/93	0.0%
	Exceeding	> 100 mg/L	93/93	100.0%
Secchi depth	Poor	< 1.0 m	0/93	0.0%
	Moderate	1.0–2.0 m	17/93	18.3%
	Good	> 2.0 m	76/93	81.7%

Results are indicative given the absence of site-specific calibration

### Algal bloom analysis

Using the 90th percentile of NDCI chlorophyll-a (121.9 µg/L) as the bloom threshold, zero of 93 lake-averaged observations exceeded this value, reflecting the fact that the threshold was computed from the full pixel-level distribution, and lake-averaged values systematically fall below pixel-level extremes. The spatial frequency of bloom occurrence across the lake surface is presented in Online Resource 5, illustrating the percentage of time each pixel exceeded the threshold concentration. This map should be interpreted as a relative indicator of bloom-prone areas within the lake rather than absolute exceedance frequencies.

### Spatial patterns

Comparison between northern and southern lake zones revealed generally homogeneous water quality conditions (Table 8). The northern zone exhibited 18.7% lower median turbidity (19.70 FNU) compared to the southern zone (24.24 FNU). Differences in other parameters were minimal (chlorophyll-a − 1.8%, TSS − 2.1%, CDOM − 6.1%, Secchi depth − 1.4%, FAI + 3.3%). The relatively small size of Bellandur Lake (1.04 km^2^) likely contributes to the observed spatial homogeneity. Temporal variations in zonal water quality are illustrated in Online Resource 6.

**Table 8:** Spatial gradient analysis comparing northern and southern lake zones

Parameter	Unit	North zone median	South zone median	Difference (%)
Chlorophyll-a (NDCI)	µg/L	70.71	71.97	− 1.8
Turbidity	FNU	19.70	24.24	− 18.7
TSS	mg/L	202.60	207.04	− 2.1
CDOM	index	82.86	88.25	− 6.1
Secchi depth	m	2.20	2.23	− 1.4
FAI	—	0.18	0.17	+ 3.3

### Pixel wise trend analysis

**Fig. 11:**
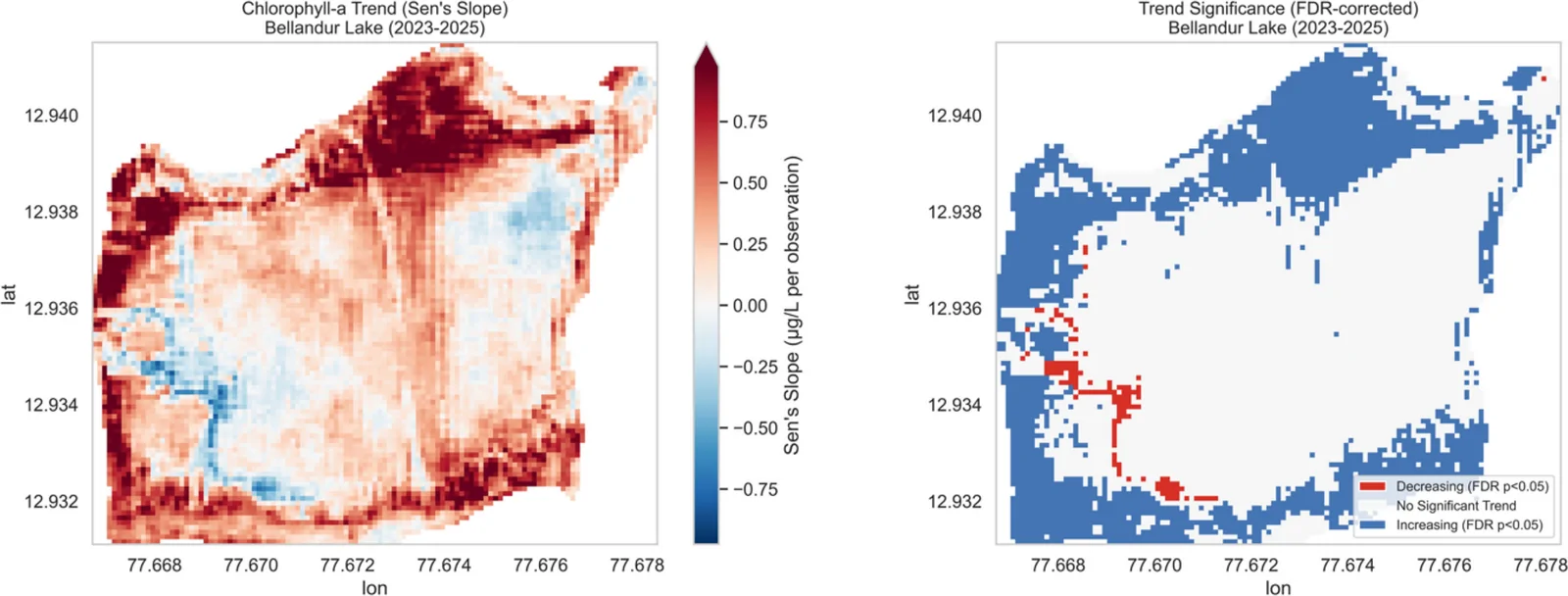
Pixel-wise Mann–Kendall trend analysis results for NDCI chlorophyll-a in Bellandur Lake (2023–2025) after Benjamini-Hochberg FDR correction. **a** Sen’s slope magnitude showing rate of change per observation. **b** FDR-corrected trend significance classification (*q* < 0.05)

Pixel-wise application of the Mann–Kendall test to NDCI chlorophyll-a time series, limited to 8829 pixels with ≥ 15 valid observations, revealed spatially heterogeneous trend patterns (Table 9). After Benjamini–Hochberg FDR correction (*q* < 0.05), significant increasing trends were detected in 2978 pixels (33.7% of analysed area) and significant decreasing trends in 131 pixels (1.5%), while 5720 pixels (64.8%) exhibited no significant trend. For comparison, uncorrected analysis at *p* < 0.05 yielded 3460 increasing and 185 decreasing pixels; the FDR correction reduced false discoveries by approximately 14%. Sen’s slope values ranged from − 0.828 to 1.538 µg/L per observation (mean 0.296, median 0.241). The predominance of increasing or stable trends corroborates the lake-averaged trend result. The spatial distribution of trend direction and magnitude is presented in Fig. 11. Given spatial autocorrelation among adjacent pixels derived from the same satellite scenes, these results are interpreted as indicative of broad spatial trend patterns rather than independently confirmed pixel-level changes.

**Table 9:** Pixel-wise trend analysis results for NDCI chlorophyll-a applying Benjamini–Hochberg FDR correction (*q* < 0.05) to address multiple testing across 8829 pixels

Trend category	Pixels	Percentage
Significant Increasing (*p *< 0.05)	2978	33.7%
Significant decreasing (*p* < 0.05)	131	1.5%
No significant trend	5720	64.8%
Total analysed	8829	100.0%

### Inter parameter correlations

Pearson correlation analysis revealed several strong relationships among water quality parameters (Table 10). The strongest positive correlation was observed between CDOM and Secchi depth (*r* = 0.962), followed by NDCI chlorophyll-a and Secchi depth (*r* = 0.854), and NDCI chlorophyll-a and CDOM (*r* = 0.808). The correlation patterns and selected relationships are illustrated in Online Resource 7.

**Table 10:** Pearson correlation coefficients among water quality parameters (*n* = 93)

Parameter	Chl-a NDCI	Chl-a (RE)	Turbidity	TSS	CDOM	Secchi	FAI
Chl-a NDCI	1.000	0.959	− 0.373	− 0.635	0.808	0.854	0.537
Chl-a (RE)		1.000	− 0.290	− 0.533	0.891	0.873	0.477
Turbidity			1.000	0.852	− 0.159	− 0.168	− 0.174
TSS				1.000	− 0.369	− 0.407	− 0.253
CDOM					1.000	0.962	0.378
Secchi depth						1.000	0.448
FAI							1.000

### Chlorophyll-a anomaly

Deviation of monthly NDCI chlorophyll-a from the long-term mean ranged from − 89.27 to + 207.66 µg/L across all pixels and time periods, with a mean anomaly of − 2.28 µg/L and standard deviation of 30.27 µg/L. Spatial patterns of monthly anomalies are presented in Online Resource 3.

### Water quality index

The composite water quality index (WQI) averaged 68.86 (median 69.94, SD 7.64) across all monthly composites, ranging from 29.50 to 93.05. As an internally normalised relative index based on the 2023–2025 dataset range, WQI values reflect relative conditions within this study period rather than absolute water quality. Monthly spatial WQI distributions are presented in Online Resource 4.

### Monthly heatmaps

Calendar heatmaps depicting monthly median values for chlorophyll-a and turbidity across all study years are presented in Online Resource 8, respectively. These visualisations highlight the seasonal patterns and interannual variability in water quality conditions.

## Discussion

### Pollution severity and trophic status

The findings indicate that Bellandur Lake exists in a state of severe and persistent eutrophication throughout the 2023–2025 study period. NDCI-based chlorophyll-a estimates (mean 71.31 µg/L) substantially exceed the hypereutrophic threshold of 25 µg/L established by the World Health Organisation and OECD, with 98.9% of observations classified as hypereutrophic based on TSI(Chl-a NDCI) (mean 71.94). These satellite-derived values should be interpreted as indicative given the absence of site-specific in situ calibration, but their magnitude and consistency across 3 years strongly suggest chronic severe eutrophication. It is noted that the NDCI-based estimates (mean ~ 71 µg/L) are substantially lower than the red-edge ratio model (mean ~ 210 µg/L); the NDCI model is considered more appropriate for this highly turbid, eutrophic environment given its explicit design for such conditions (Mishra and Mishra 2012; Matthews and Bernard 2013).

The 23.6-unit gap between TSI(Chl-a) = 71.94 and TSI(Secchi) = 48.36 reflects the CDOM-dominated optical environment of Bellandur Lake rather than a genuine contradiction. CDOM from sewage, industrial effluents, and decomposing organic matter strongly attenuates light at visible wavelengths, decoupling Secchi depth from phytoplankton biomass. Because underwater light penetration fundamentally controls aquatic photosynthetic conditions and optical water clarity, Secchi-based trophic interpretation becomes unreliable in optically complex systems such as Bellandur Lake (Kirk 1994). This finding is consistent with highly polluted tropical urban lakes where multiple optically active components co-dominate the light field (Greb et al. 2018; Huang et al. 2025). Averaging the two TSI components, as sometimes applied to systems where both indicators reliably reflect trophic state, is not appropriate here and would yield a misleadingly lower classification.

The assessment against Central Pollution Control Board standards for total suspended solids indicates 100% exceedance of the class D limit (100 mg/L) across all observations. It is important to emphasise that, given the absence of site-specific in situ calibration for the Nechad et al. (2010) TSS algorithm, the absolute TSS concentrations reported here are likely overestimated for reasons outlined in the “Total suspended solids” section and “Algorithm performance and validation considerations” section. The compliance finding should therefore be interpreted as indicative of chronic severe particulate loading rather than as a formally validated regulatory determination. In situ measurement would be required to confirm regulatory non-compliance with the precision needed for enforcement purposes (Chapman 2022; Jaywant and Arif 2024). Notwithstanding this caveat, the consistently high satellite-derived TSS values across all 93 observations, combined with well-documented field accounts of chronic sewage and effluent loading at Bellandur Lake (Ramachandra and Kumar 2008; Das et al. 2023; Mampilamthoda and Chinnasamy 2025), strongly support the qualitative conclusion that TSS standards are persistently exceeded. Elevated TSS concentrations impair light penetration, reduce dissolved oxygen through organic matter decomposition, and transport adsorbed contaminants (Bilotta and Brazier 2008). Similar patterns of chronic TSS exceedance have been documented in other Indian urban lakes receiving inadequately treated sewage (Tibebe et al. 2023; Ramachandra et al. 2023).

### Temporal trends and potential drivers

The significant decreasing trends in turbidity (72.5% reduction from 2023 to 2025) and total suspended solids (31.3% reduction) are notable findings. The use of NDCI-based chlorophyll-a as the primary variable revealed a significant increasing trend (*p* = 0.041) that was not apparent using the red-edge ratio model (*p* = 0.068), highlighting the importance of algorithm selection for trend detection in hypereutrophic systems. This finding underscores the recommendation of Salls et al. (2024) and Joffre et al. (2026) that algorithm choice should be explicitly justified and sensitivity-tested in hypereutrophic monitoring applications. The increasing chlorophyll-a trend is consistent with satellite monitoring studies of eutrophic lakes demonstrating widespread increases in phytoplankton biomass across Asian urban lakes (Ho et al. 2019; Wang et al. 2024), and aligns with observed trajectories in other intensively urbanised South Asian catchments (Anand et al. 2024).

Several factors may contribute to the observed particulate reductions. The Bangalore Water Supply and Sewerage Board has implemented sewage treatment capacity improvements during the study period, potentially reducing inorganic particulate loading (Tibebe et al. 2023). However, the concurrent significant increase in the floating algae index (117% increase, *τ* = 0.419, *p* < 0.001) and significant increase in NDCI chlorophyll-a suggest that nutrient loading remains sufficient to sustain and expand algal productivity. This decoupling between suspended solids and algal biomass trends is consistent with the internal loading phenomenon whereby phosphorus accumulated in sediments continues to fuel primary production even as external particulate loading decreases (Søndergaard et al. 2003; Jeppesen et al. 2005; Tammeorg et al. 2023).

The strong FAI increasing trend (Sen’s slope = 0.002 per observation) is particularly notable. Cyanobacteria capable of buoyancy regulation, common in tropical hypereutrophic lakes, are increasingly documented to respond positively to warming temperatures and stable stratification (Huisman et al. 2018; Paerl et al. 2016). The co-occurrence of decreasing turbidity with increasing FAI and chlorophyll-a is consistent with a shift toward phytoplankton-dominated optical properties as inorganic suspended matter declines.

### Seasonal dynamics and monsoon influence

The significant differences between pre-monsoon and post-monsoon water quality conditions provide insights into seasonal pollution dynamics. The 67.4% turbidity reduction and 20.6% TSS reduction during post-monsoon periods suggest that monsoon flushing temporarily dilutes and exports particulate matter. Similar seasonal patterns have been documented in other monsoonal lake systems (Zhang et al. 2014; Seth et al. 2025). The 51.4% FAI reduction during post-monsoon may reflect physical disruption of surface algal accumulations by monsoon precipitation and wind. However, the absence of significant chlorophyll-a change between seasons (− 6.8%, *p* = 0.278) confirms that nutrient availability remains sufficient to sustain algal productivity regardless of hydrological flushing, consistent with chronic nutrient enrichment.

The limited data availability during monsoon months represents an inherent constraint of optical remote sensing in monsoonal climates, widely acknowledged in studies of Indian water bodies (Uday et al. 2025; Seth et al. 2025). The five JJA observations available should be treated cautiously in any seasonal interpretation.

### Floating algae and surface accumulation

The strongly significant increasing FAI trend (*τ* = 0.419, *p* < 0.001) is notable given the association between surface algal accumulation and the foam formation events that have brought international attention to Bellandur Lake (Das et al. 2023). The FAI algorithm detects elevated near-infrared reflectance relative to a spectral baseline, indicating floating algae, algal mats, or organic surface films (Hu 2009). The increasing FAI trend concurrent with increasing NDCI chlorophyll-a and stable Secchi depth suggests possible shifts in algal community composition toward buoyancy-regulating species or increased accumulation of surface organic matter. Cyanobacterial dominance is well-documented in hypereutrophic tropical lakes, and climate change is expected to further exacerbate harmful cyanobacterial blooms through increased water temperatures and altered precipitation patterns (Huisman et al. 2018; Paerl et al. 2016). Surface accumulations can lead to elevated cyanotoxin concentrations posing risks to human and animal health (Chorus and Welker 2021). Satellite-based cyanobacteria monitoring frameworks are increasingly being developed to support such public health surveillance (Maniyar et al. 2023), underscoring the broader management relevance of the FAI increasing trend detected here.

### Spatial patterns

The minimal differences between northern and southern lake zones (0.4 to 18.7% range) suggest relatively homogeneous conditions. The 18.7% lower turbidity in the northern zone may reflect proximity to outflow structures or localised differences in sediment resuspension patterns. The small lake size (1.04 km^2^) facilitates wind-driven mixing that homogenises spatial gradients (Toffolon et al. 2020). FDR-corrected pixel-wise analysis revealed that 33.7% of the lake area exhibited significant increasing NDCI chlorophyll-a trends (compared to 21.2% in uncorrected analysis), with only 1.5% showing decreasing trends. The predominance of stable or increasing trends indicates absence of widespread improvement in algal productivity. Similar spatial heterogeneity has been observed in satellite assessments of other eutrophic lakes (Ho et al. 2019; Wang et al. 2024).

### Correlation patterns

The strong CDOM–Secchi depth correlation (*r* = 0.962) partly reflects the shared B2/B3 spectral input in their respective algorithms rather than purely environmental co-variation. Similarly, the turbidity–TSS correlation (*r* = 0.852) partly reflects shared dependence on the B4 red band. These mathematical dependencies should be acknowledged when interpreting the correlation matrix. The positive NDCI chlorophyll-a–Secchi depth relationship (*r* = 0.854) is attributable to CDOM-dominated light attenuation: in systems where CDOM rather than phytoplankton primarily controls transparency, the expected negative algal biomass–transparency relationship is modified (Brezonik et al. 2015; Huang et al. 2025). The negative correlations between NDCI chlorophyll-a and both turbidity (*r* = − 0.373) and TSS (*r* = − 0.635) suggest that elevated inorganic suspended sediment during high-turbidity periods suppresses light availability for algal growth, or alternatively, that different pollution sources dominate during high turbidity versus high algal productivity periods.

### Algorithm performance and validation considerations

The empirical algorithms employed were developed and validated for other water bodies and applied without site-specific calibration or validation for Bellandur Lake. The absolute values of derived parameters should therefore be interpreted as indicative estimates of relative spatial and temporal patterns rather than confirmed absolute concentrations. Direct comparison with in situ measurements from other studies or with regulatory thresholds based on field measurements should be made with explicit acknowledgement of this limitation (Odermatt et al. 2012; Greb et al. 2018; Jaywant and Arif 2024). The suitability of empirical algorithms is fundamentally constrained by the principle of optical transferability: algorithms calibrated in one optical environment may not accurately retrieve the same parameter in a different optical environment, even if the parameter of interest is nominally the same (Pahlevan et al. 2022; Salls et al. 2024). Bellandur Lake presents an unusually challenging optical environment characterised by the simultaneous co-occurrence of hypereutrophic phytoplankton concentrations, elevated inorganic suspended sediment, high CDOM loading from sewage and organic decomposition, and episodic surface foam and floating algal accumulations. This multi-constituent complexity is outside the calibration envelope of most of the algorithms applied here, which were developed either for coastal mineral-dominated systems (Nechad et al. 2010; Dogliotti et al. 2015) or for productive but less optically complex inland lakes (Binding et al. 2007; Brezonik et al. 2015). The fundamental challenge in such environments is that spectral reflectance in any given band is a composite of contributions from all optically active constituents, and single-constituent empirical algorithms cannot fully disentangle these contributions without additional spectral information or a priori optical constraints (Odermatt et al. 2012; Pahlevan et al. 2022). Specific algorithm-level concerns are as follows. For chlorophyll-a, the NDCI model (Mishra and Mishra 2012) was specifically designed for turbid productive waters and has been validated across diverse hypereutrophic inland systems with root mean square errors typically ranging from 20 to 40% (Matthews and Bernard 2013; Zhang et al. 2014; Salls et al. 2024). The normalised difference form reduces (but does not eliminate) sensitivity to non-algal particles and CDOM compared to simple band ratio algorithms. However, at very high chlorophyll concentrations, NDCI can approach saturation as B5 and B4 reflectance both become strongly influenced by phytoplankton absorption, reducing index sensitivity a limitation documented in hypereutrophic systems by Salls et al. (2024). Additionally, the presence of surface foam at Bellandur Lake introduces spectrally anomalous reflectance signatures that cannot be fully masked by standard cloud screening procedures and may bias lake-average chlorophyll estimates in ways that are difficult to quantify without concurrent optical measurements. For turbidity, the Dogliotti et al. (2015) algorithm was validated across 1 to 1000 FNU with mean absolute percentage errors below 30%; however, its calibration dataset was dominated by coastal mineral turbidity, and performance under mixed turbidity sources (phytoplankton, sediment, foam) as found in Bellandur Lake is uncertain. For TSS, overestimation due to co-occurring optically active constituents is expected and documented in the “Total suspended solids” section above. For CDOM and Secchi depth, the shared B2/B3 spectral dependence creates correlated uncertainties discussed in the “Secchi disk depth” section. Regarding the Secchi depth map for May 2024, inspection of the imagery for that date revealed that masking patterns in the Secchi depth product differ from other parameters due to the sensitivity of the B2/B3 ratio to thin cloud and haze that may not be fully masked by the cloud probability threshold. This is an acknowledged limitation of B2/B3-based algorithms in near-cloud conditions and was not exclusive to that date, though most other scenes were unaffected. Despite these algorithm-level limitations, temporal trends and broad spatial patterns in this study are less sensitive to systematic calibration biases than absolute concentrations, because systematic offsets tend to affect all observations proportionally. The consistency of directional findings across multiple independent parameters increasing chlorophyll-a and FAI concurrent with decreasing turbidity and TSS strengthens confidence in the overall interpretation. Future studies incorporating concurrent in situ sampling would substantially improve confidence in absolute accuracy and enable site-specific algorithm calibration, which remains the essential next step for converting this monitoring framework into a validated water quality product.

### Management implications

The findings indicate that despite apparent reductions in particulate pollution (turbidity and TSS), Bellandur Lake remains severely degraded with no improvement in eutrophication status; indeed, NDCI chlorophyll-a and FAI both exhibit significant increasing trends. The persistence of hypereutrophic conditions despite decreasing particulate loading suggests that nutrient management must remain the primary focus of restoration efforts. External nutrient loading from inadequately treated sewage requires continued infrastructure investment, while the potential contribution of internal nutrient loading from contaminated sediments may necessitate sediment management interventions (Huser et al. 2016; Tammeorg et al. 2023). The strongly significant increasing FAI trend suggests that surface accumulation events may intensify without intervention targeting underlying nutrient enrichment. Remote sensing-based monitoring as demonstrated here provides a cost-effective approach for tracking management effectiveness over time and detecting changes across the entire lake surface.

## Conclusion

This study assessed water quality dynamics in Bellandur Lake using Sentinel-2 satellite imagery from January 2023 to December 2025, processing 100 images to derive six water quality parameters across 93 valid observations. All satellite-derived values are interpreted as indicative of relative spatial and temporal patterns given the absence of site-specific in situ validation. The lake exhibited severely degraded conditions throughout the study period, with mean NDCI-based chlorophyll-a of 71.31 µg/L (median 70.24 µg/L) and TSI(Chl-a) of 71.94, indicating persistent hypereutrophic status in 78.5% of observations. Mann–Kendall trend analysis, using NDCI as the primary chlorophyll-a variable, identified significant increasing trends in chlorophyll-a (*τ* = 0.144, *p* = 0.041) and floating algae index (*τ* = 0.419, *p* < 0.001) alongside significant decreasing trends in turbidity (*p* < 0.001) and total suspended solids (*p* = 0.001). FDR-corrected pixel-wise trend analysis identified 33.7% of the lake area exhibiting significant increasing chlorophyll-a trends. The divergence between TSI(Chl-a) and TSI(Secchi) (approximately 23.6 units) reflects CDOM-dominated light attenuation decoupling Secchi depth from trophic state; trophic classification was accordingly based exclusively on TSI(Chl-a). Complete non-compliance with CPCB standards was documented, with 100% of observations exceeding permissible TSS limits and 98.9% classified as hypereutrophic.

These findings indicate that, consistent with relative spatiotemporal patterns detectable from satellite remote sensing, eutrophication and surface algal accumulation appear to be intensifying in Bellandur Lake across the 2023–2025 study period, with NDCI-derived chlorophyll-a and floating algae index both exhibiting statistically significant upward trends. These results are considered indicative given the absence of site-specific algorithm validation; absolute concentrations and regulatory compliance assessments reported here should not be treated as confirmed analytical determinations until concurrent in situ measurement and algorithm calibration are performed. Subject to this caveat, the findings suggest that nutrient management remains critical for any restoration strategy and that reductions in particulate loading have not translated into improvements in eutrophication status. The study establishes a methodologically transparent baseline employing NDCI as the primary chlorophyll-a variable, FDR correction for multiple testing, and explicit acknowledgement of algorithm transferability limitations that can serve as a reproducible framework for tracking water quality trajectories in Bellandur Lake and comparable hypereutrophic urban lake systems in South Asia. Future research should incorporate concurrent in situ sampling for algorithm calibration, investigate linkages between water quality parameters and specific pollution sources, and evaluate the potential of more advanced retrieval approaches including machine learning methods trained on local optical properties to improve absolute accuracy in this optically complex environment.

## Supplementary Information

Below is the link to the electronic supplementary material.

ESM 1 (PDF 1.01 MB)

ESM 2 (PDF 1.03 MB)

ESM 3 (PDF 1.16 MB)

ESM 4 (PDF 811 KB )

ESM 5 (PDF 379 KB)

ESM 6 (PDF 382 KB)

ESM 7 (PDF 369 KB)

ESM 2 (PDF 370 KB)

## Data Availability

All data and code supporting the findings of this study are available in a public GitHub repository at (https://github.com/satyamspace/Hypereutrophic-Lake-Remote-Sensing). Sentinel 2 satellite imagery is publicly accessible through Google Earth Engine (https://earthengine.google.com/).

## Declarations

### Clinical trial number

This is not applicable.
