# Supplementary material for: Spatiotemporal monitoring of water quality degradation and trophic state in a severely polluted tropical urban lake in Southern India
Source: Environ Sci Pollut Res Int. 2026 Oct 23;33(38):17686–707. doi: 10.1007/s11356-026-38316-4 (PMC13739951; doi:10.1007/s11356-026-38316-4)
Supplement: Supplementary file 8 [file 11356_2026_38316_MOESM8_ESM.pdf]

Environmental Science and Pollution Research

Spatiotemporal monitoring of water quality degradation and trophic state in a severely polluted tropical urban lake in southern India

Satyam Shah
School of Geography, Geology and the Environment, University of Leicester, United Kingdom

Email: satyamshah444@gmail.com

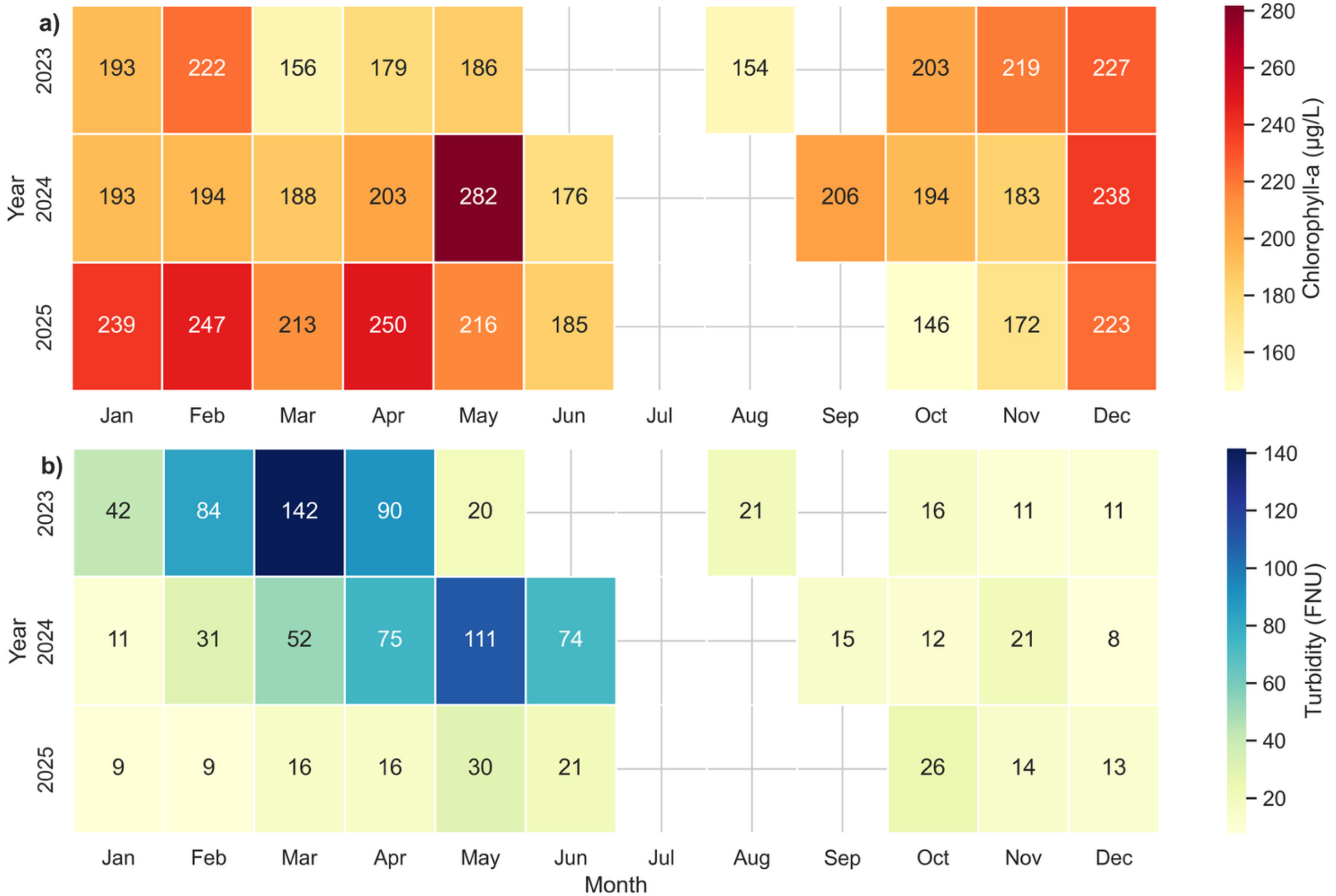

Online Resource 8. Calendar heatmaps showing monthly median values for (a) NDCI chlorophyll-a (µg/L) and (b) turbidity (FNU) in Bellandur Lake from January 2023 to December 2025.
